# Association Between Retinal Pathology and Hearing in Older Adults

**DOI:** 10.1002/oto2.99

**Published:** 2023-12-13

**Authors:** Zahra N. Sayyid, Armaan F. Akbar, Kening Jiang, Jacob M. Pogson, Nicholas S. Andresen, Jennifer A. Deal, Bryan K. Ward

**Affiliations:** ^1^ Department of Otolaryngology–Head and Neck Surgery Johns Hopkins School of Medicine Baltimore Maryland USA; ^2^ Johns Hopkins Bloomberg School of Public Health Cochlear Center for Hearing and Public Health Baltimore Maryland USA; ^3^ Department of Epidemiology Johns Hopkins Bloomberg School of Public Health Baltimore Maryland USA; ^4^ Department of Neurology Johns Hopkins School of Medicine Baltimore Maryland USA; ^5^ Department of Neurology Royal Prince Alfred Hospital Camperdown New South Wales Australia; ^6^ Disability Health Research Center Johns Hopkins University Baltimore Maryland USA

**Keywords:** hearing loss, National Health and Nutritional Examination Survey, retinal microvascular changes, retinal pathology

## Abstract

We investigated the association between retinal microvascular changes and hearing loss based on the hypothesis that both may result from shared microvascular pathology. Data from 536 older adults from the National Health and Nutritional Examination Survey 2005 to 2006 including sociodemographic and health characteristics, pure‐tone hearing thresholds, and retinal pathologies were collected and analyzed. Associations between retinal and hearing pathologies were modeled with multivariable‐adjusted linear regressions. 75% of participants had hearing loss and 15% of participants had retinopathy. The association between retinopathy, microaneurysms, and blot hemorrhages with better speech‐frequency pure tone average was −2.81 (95% confidence interval [CI]: −5.72 to 0.10), −4.75 (95% CI: −8.73 to −0.78), and −5.34 (95% CI: −8.68 to −2.00), respectively. The presence of retinopathy, microaneurysms, and blot hemorrhages was inversely associated with hearing loss. Further studies are needed to better understand the potential relationship between microvascular pathologies of the eye and ear.

Age‐related hearing loss is prevalent and associated with several adverse health outcomes,^1^, ^2^, ^3^, ^4^ including cognitive decline.^5^ Vascular pathologies could contribute to hearing loss through impaired cochlear blood supply.^6^ Visualization of cochlear microcirculation using well‐established clinical imaging modalities is challenging due to insufficient spatial resolution or contrast.^7^, ^8^, ^9^, ^10^ The retinal microvasculature, however, can be imaged noninvasively to measure changes in cerebral microvasculature,^11^, ^12^, ^13^, ^14^ changes that we hypothesize could apply to the enshrouded cochlear microvasculature. In this study, we use a nationally representative sample from the National Health and Nutritional Examination Survey (NHANES) to investigate the association between retinal microvascular changes and hearing loss based on the hypothesis that both result from shared microvascular pathology.

## Methods

Adults aged ≥70 years from the 2005 to 2006 cycle of NHANES were studied. Pure‐tone hearing thresholds in decibels (dB) hearing level were averaged to create speech‐frequency (0.5‐4 kHz), low‐frequency (0.5‐2 kHz), and high‐frequency (4‐8 kHz) pure‐tone averages (PTAs) in the worse‐hearing ear, with greater values indicating worse hearing. Hearing loss was defined as PTA>25 dB in the worse hearing ear. The presence of retinal pathologies, including retinopathy, arteriovenous nicking, focal arteriolar narrowing, microaneurysms, and blot hemorrhages for the worse‐seeing eye, were identified from graded fundus photographs. Sociodemographic characteristics, including age, sex, race, education, smoking history, and occupational exposure to loud noise, were collected in addition to health characteristics, including hypertension, diabetes, stroke, and body mass index.

Means and standard deviations or frequencies and percentages of demographics and health characteristics were examined. Multivariable‐adjusted linear regression was used to model the associations and was stratified by diabetes status. A 2‐sided *P* < .05 was considered statistically significant. Statistical analyses were conducted using Stata version 17.0 (StataCorp LLC). The study protocol was approved by the National Center for Health Statistics Institutional Review Board; all participants provided written informed consent.

## Results

**Figure 1:**
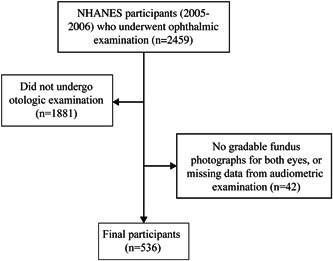
Flow chart of selection of study participants. NHANES, National Health and Nutritional Examination Survey.

The final sample comprised 536 participants (Figure 1, Table 1). The prevalence of hearing loss was $\frac{402}{536}$ (75.0%), and the mean speech‐frequency PTA was 37.4 dB HL (standard deviation: 15.3) in the worse‐hearing ear. The presence of retinal pathology ranged from 4.1% (focal arteriolar narrowing) to 17.9% (arteriovenous nicking). Participant characteristics by retinopathy in the worse eye are described in Supplemental Table S1, available online. While $\frac{81}{536}$ participants (15.1%) had retinopathy, the distributions of speech‐frequency, low‐frequency, and high‐frequency PTAs were similar between those with and without retinopathy.

**Table 1:** Baseline Characteristics by the Presence of Hearing Loss in Worse Ear

	Total cohort (n = 536)	No hearing Loss (n = 134)	Hearing loss (n = 402)
Age, y			
70‐75	215 (40.1%)	78 (58.2%)	137 (34.1%)
75‐80	139 (25.9%)	33 (24.6%)	106 (26.4%)
80‐85	123 (22.9%)	14 (10.4%)	109 (27.1%)
85 and older	59 (11.0%)	9 (6.7%)	50 (12.4%)
Female sex	247 (46.1%)	75 (56.0%)	172 (42.8%)
Race			
Non‐Hispanic Black	76 (14.2%)	30 (22.4%)	46 (11.4%)
Non‐Hispanic White	401 (74.8%)	91 (67.9%)	310 (77.1%)
Other	59 (11.0%)	13 (9.7%)	46 (11.4%)
Education level			
Less than high school	191 (35.6%)	32 (23.9%)	159 (39.6%)
High school or equivalent	155 (28.9%)	37 (27.6%)	118 (29.4%)
Above high school	190 (35.4%)	65 (48.5%)	125 (31.1%)
Ever smoker	305 (56.9%)	71 (53.0%)	234 (58.2%)
Loud noise exposure	219 (40.9%)	38 (28.4%)	181 (45.0%)
Hypertension	380 (70.9%)	98 (73.1%)	282 (70.1%)
Diabetes status			
No	199 (37.1%)	51 (38.1%)	148 (36.8%)
Prediabetes	202 (37.7%)	49 (36.6%)	153 (38.1%)
Diabetes	135 (25.2%)	34 (25.4%)	101 (25.1%)
Stroke	54 (10.1%)	12 (9.0%)	42 (10.4%)
Body mass index (kg/m^2^)	27.7 ± 5.3	27.8 ± 5.9	27.6 ± 5.1
Retinopathy	81 (15.1%)	19 (14.2%)	62 (15.4%)
Arteriovenous nicking	96 (17.9%)	21 (15.7%)	75 (18.7%)
Focal arteriolar narrowing	22 (4.1%)	4 (3.0%)	18 (4.5%)
Microaneurysms	64 (11.9%)	16 (11.9%)	48 (11.9%)
Blot hemorrhages	60 (11.2%)	12 (9.0%)	48 (11.9%)

Differences in worse‐ear speech‐, low‐, and high‐frequency PTAs by worse‐eye retinal signs after adjusting for age, sex, race, education, smoking, and occupational exposure to noise, stratified by diabetes status, are shown in Table 2. Associations with better speech‐frequency PTA were found among those with diabetes for microaneurysms (−7.87, 95% CI: −15.36 to −0.39) and blot hemorrhages (−8.28, 95% CI: −15.88 to −0.67), and among those without diabetes for blot hemorrhages (−6.13, 95% CI: −11.83 to −0.45). The associations with better low‐frequency PTA among those with diabetes were −7.42 (−14.18 to −2.83) for microaneurysms and −8.67 (−16.81 to –0.52) for blot hemorrhages, and those without diabetes were −4.15 (95% CI: −7.47 to −0.83) for retinopathy and −7.45 (−12.37 to −2.53) for blot hemorrhages. There were no associations between better high‐frequency PTA and retinal pathology. Among those without diabetes, however, the association with worse high‐frequency PTA was 6.32 (0.66‐11.97) for focal arteriolar narrowing. There was no significant association between arteriovenous nicking and PTAs.

**Table 2:** Multivariable‐Adjusted Linear Models for Speech‐FrequeNcy, Low‐Frequency, and High‐frequency PTAs in Worse‐Hearing Ear by Retinal Signs in Worse Eye, Stratified by Diabetes Status

	Speech‐frequency PTA		Low‐frequency PTA		High‐frequency PTA	
	Coefficient (95% CI)	*P*	Coefficient (95% CI)	*P*	Coefficient (95% CI)	*P*
Retinopathy						
Diabetes	−3.43 (−9.54 to 2.68)	.25	−3.57 (−10.11 to 2.96)	.26	−3.78 (−12.18 to 4.62)	.35
No diabetes	−3.33 (−7.18 to 0.53)	.09	−4.15 (−7.47 to −0.83)	.02*	−0.58 (−6.96 to 5.81)	.85
Arteriovenous nicking						
Diabetes	1.63 (−7.73 to 11.00)	.72	1.61 (−7.50 to 10.72)	.71	5.69 (−6.27 to 17.65)	.33
No diabetes	0.92 (−1.86 to 3.71)	.49	1.34 (−2.08 to 4.76)	.42	0.47 (−1.97 to 2.91)	.69
Focal arteriolar narrowing						
Diabetes	−2.90 (−29.47 to 23.67)	.82	3.10 (−21.32 to 27.52)	.79	−3.45 (−25.72 to 18.82)	.75
No diabetes	3.73 (−3.30 to 10.77)	.28	3.62 (−4.02 to 11.26)	.33	6.32 (0.66 to 11.97)	.03*
Microaneurysms						
Diabetes	−7.87 (−15.36 to −0.39)	.04*	−7.42 (−14.18 to −0.65)	.03*	−11.86 (−24.32 to 0.60)	.06
No diabetes	−2.29 (−.645 to 2.06)	.28	−2.83 (−7.39 to 1.74)	.21	−2.71 (−10.78 to 5.36)	.49
Blot hemorrhages						
Diabetes	−8.28 (−15.88 to −0.67)	.04*	−8.67 (−16.81 to −0.52)	.04*	−3.67 (−16.30 to 8.97)	.55
No diabetes	−6.13 (−11.82 to −0.45)	.04*	−7.45 (−12.37 to −2.53)	.01*	−3.19 (−13.30 to 6.91)	.51

Models adjusted for age, sex, race, education, smoking, and occupational exposure to noise.

Abbreviations: CI, confidence interval; PTA, pure tone average.

* *P* < .05.

## Discussion

This study found an association of retinopathy, microaneurysms, and blot hemorrhages with better hearing; in other words, more retinal pathologies were associated with less hearing loss. This association was significant for low‐frequency PTA across all 3 retinal pathologies and borderline for the association between retinopathy and speech‐frequency PTA. This seemed to occur regardless of the presence of diabetes. Interestingly, there were no significant associations between retinal pathology and better high‐frequency PTA. In those without diabetes, however, focal arteriolar narrowing was associated with worse high‐frequency PTA.

This study adds to the small body of literature investigating the relationship between retinal microvascular abnormalities and hearing loss. In the Blue Mountains hearing study, Liew et al performed retinal photography and PTA on individuals aged 54 and older. They found an association between retinopathy and low‐frequency hearing loss in women only.^15^ More recently, Kim et al studied patients aged 45 to 64 and found that retinopathy was significantly associated with better rather than worse higher‐frequency hearing.^16^ The difference in findings between our study and prior studies may be attributed to differences in the demographics of our cohort. Prior work has shown that the relationship between retinal pathology and hearing loss can be elucidated by examining the degree of microvascular pathology, with only those with more severe maculopathy or diabetic retinopathy being associated with worse hearing loss.^17^, ^18^, ^19^ Our relatively healthier population of adults 70 years and older showed a high incidence of hearing loss but a lower incidence of other comorbidities, whereas 75% of participants had hearing loss, only 25% had diabetes, and less than 20% had retinal pathology. The degree of retinal and, thus, microvascular pathology in our cohort may have been milder than in previously studied cohorts, preventing our ability to support the hypothesis of a common microvascular pathology contributing to both retinal disease and hearing loss.^20^

Alternatively, the inverse association between retinal pathologies and low‐ and speech‐frequency hearing loss identified here suggests a potential protective relationship between the visual and auditory systems, where the impairment of one system is associated with better performance in the other system.

Recent studies have found an association between metformin, a medication commonly used to treat diabetes, and reduced risk of sensorineural hearing loss.^21^ We found, however, protective relationships in the groups with and without diabetes. Future studies can help further elucidate whether this type of sensory compensation can occur later in life.^22^

## Conclusion

We found an inverse association between retinopathy, microaneurysms, and blot hemorrhages with hearing loss, adding to the literature examining the association between retinal abnormalities and hearing loss in older adults. Further epidemiologic studies are needed to understand better the potential relationship between microvascular pathologies of the eye and ear.

## Author Contributions

**Zahra N. Sayyid**, analyzed data and wrote the initial manuscript draft; **Armaan F. Akbar**, analyzed data and wrote the initial manuscript draft; **Kening Jiang**, designed the study and analyzed data; **Jacob M. Pogson**, designed the study; **Nicholas S. Andresen**, designed the study; **Jennifer A. Deal**, designed the study; **Bryan K. Ward**, designed the study. All authors contributed to the final draft of the manuscript.

## Disclosures

### Competing interests

The authors declare that there is no conflict of interest.

### Funding sources

This research received no specific grant from any funding agency in the public, commercial, or not‐for‐profit sectors.

## Supporting information

Supporting information.
