# Supplementary material for: Association Between Retinal Pathology and Hearing in Older Adults
Source: OTO Open. 2023 Dec 13;7(4):e99. doi: 10.1002/oto2.99 (PMC10718468; doi:10.1002/oto2.99)
Supplement: Supplementary file 1 — Supporting information. [file OTO2-7-e99-s001.docx]

Supplemental Table 1. Baseline characteristics by the presence of retinopathy in worse eye

|  | Total Cohort  (n = 536) | No Retinopathy  (n = 455) | Retinopathy  (n = 81) |
| --- | --- | --- | --- |
| Age (years) |  |  |  |
| 70-75 | 215 (40.1%) | 185 (40.7%) | 30 (37.0%) |
| 75-80 | 139 (25.9%) | 118 (25.9%) | 21 (25.9%) |
| 80-85 | 123 (22.9%) | 105 (23.1%) | 18 (22.2%) |
| 85 and older | 59 (11.0%) | 47 (10.3%) | 12 (14.8%) |
| Female sex | 247 (46.1%) | 215 (47.3%) | 32 (39.5%) |
| Race |  |  |  |
| Non-Hispanic Black | 76 (14.2%) | 56 (12.3%) | 20 (24.7%) |
| Non-Hispanic White | 401 (74.8%) | 353 (77.6%) | 48 (59.3%) |
| Other | 59 (11.0%) | 46 (10.1%) | 13 (16.0%) |
| Education level |  |  |  |
| Less than high school | 191 (35.6%) | 154 (33.8%) | 37 (45.7%) |
| High school or equivalent | 155 (28.9%) | 139 (30.5%) | 16 (19.8%) |
| Above high school | 190 (35.4%) | 162 (35.6%) | 28 (34.6%) |
| Ever smoker | 305 (56.9%) | 259 (56.9%) | 46 (56.8%) |
| Loud noise exposure | 219 (40.9%) | 179 (39.3%) | 40 (49.4%) |
| Hypertension | 380 (70.9%) | 312 (68.6%) | 68 (84.0%) |
| Diabetes status |  |  |  |
| No | 199 (37.1%) | 177 (38.9%) | 22 (27.2%) |
| Prediabetes | 202 (37.7%) | 182 (40.0%) | 20 (24.7%) |
| Diabetes | 135 (25.2%) | 96 (21.1%) | 39 (48.1%) |
| Stroke | 54 (10.1%) | 40 (8.8%) | 14 (17.3%) |
| Body mass index (kg/m2) | 27.7 ± 5.3 | 27.4 ± 5.3 | 29.0 ± 5.3 |
| Speech PTA (0.5-4kHz) |  |  |  |
| No hearing loss | 134 (25.0%) | 115 (25.3%) | 19 (23.5%) |
| Hearing loss | 402 (75.0%) | 340 (74.7%) | 62 (76.5%) |
| Low PTA (0.5-2kHz) |  |  |  |
| No hearing loss | 229 (42.7%) | 194 (42.6%) | 35 (43.2%) |
| Hearing loss | 307 (57.3%) | 261 (57.4%) | 46 (56.8%) |
| High PTA (4-8kHz) |  |  |  |
| No hearing loss | 15 (2.8%) | 13 (2.9%) | 2 (2.5%) |
| Hearing loss | 521 (97.2%) | 442 (97.1%) | 79 (97.5%) |

PTA = pure tone average
